# Modulation of physiological and biochemical traits of two genotypes of *Rosa damascena* Mill. by SiO_2_-NPs under *In vitro* drought stress

**DOI:** 10.1186/s12870-022-03915-z

**Published:** 2022-11-18

**Authors:** Hanifeh Seyed Hajizadeh, Sahar Azizi, Farzad Rasouli, Volkan Okatan

**Affiliations:** 1 Department of Horticulture, Faculty of Agriculture, University of Maragheh, Maragheh, 55136-553 Iran; 2 Department of Horticulture, Faculty of Agriculture, Eskisehir Osmangazi University, Eskisehir, Turkey

**Keywords:** Damask, Micropropagation, Polyethylene glycol, Nanoparticles, Abiotic stress, Antioxidative status

## Abstract

**Background:**

Drought is a major abiotic stress that restricts plant growth and efficiency although some nutrients such as silicon improve drought tolerance by regulating the biosynthesis and accumulating some osmolytes. In this regard, a completely randomized factorial design was performed with three factors including two genotypes (‘Maragheh’ and ‘Kashan’), three concentrations of silicon dioxide nanoparticles (SiO_2_-NPs) (0, 50, and 100 mg L^− 1^), and five concentrations of PEG (0, 25, 50, 75, and 100 g L^− 1^) with three replications.

**Results:**

The findings showed that drought stress decreased protein content and it was improved by SiO_2_-NPs, so the genotype of ‘Maragheh’ treated with 100 mg L^− 1^ SiO_2_-NPs had the highest protein content. Under severe drought stress, had a higher membrane stability index (MSI) than ‘Kashan’, and the ‘Maragheh’ explants subjected to 100 mg L^− 1^ SiO_2_-NPs exhibited the uppermost MSI. The explants supplemented with 100 mg L^− 1^ SiO_2_-NPs sustained their photosynthetic parameters more in comparison with other treatments under drought stress conditions and as well as 100 mg L^− 1^ SiO_2_-NPs showed higher content of protein and proline of ‘Maragheh’ than ‘Kashan’. Drought stress reduced *Fm*, *Fv/Fm*, and *Fv*, while SiO_2_-NPs treatment enhanced these parameters. SiO_2_-NPs also improved water deficit tolerance by enhancing the activity of antioxidant enzymes such as catalase (CAT), peroxidase (POD), guaiacol peroxidase (GPX), and superoxide dismutase (SOD) and reducing lipid peroxidation and H_2_O_2_ concentration.

**Conclusions:**

According to the findings, the genotype ‘Maragheh’ was more tolerance to drought stress than ‘Kashan’ by improving water balance, antioxidant enzyme activities, and membrane stability as it was obtained from the unpublished previous evaluation in in vivo conditions and we concluded based on these results, in vitro culture can be used for drought screening in Damask rose plants. The results of the current study revealed that the induced drought stress by polyethylene glycol (PEG) in two Damask rose genotypes was ameliorated with SiO_2_-NPs and the tolerance genotypes were better than the sensitive ones in response to SiO_2_-NPs treatment.

## Background

Rose is one of the most important commercial flowers among ornamentals and it is very popular as an ornamental garden plant, cut flower, potted plant, and also medicinal plant [1]. *Rosa damascena* Miller var*.* trigintipetala Dieck is a pink rose that is a hybrid called *Rosa × damascena* [2]. It has been suggested that Damask rose has been developed in Iran by the hybridization of *R. moschata* Benth., *R. gallica* L., and *R. feldschenkoana* Regel, so its origin is Iran [3, 4] where its essential oil has high quality because of the desired climatic and growing conditions [5]. It can be propagated by all common vegetative methods such as the sucker, cutting, budding, and grafting techniques [6]. Since the mentioned techniques are time-consuming, the use of micropropagation can be useful for producing a lot of genetically similar plants at the same time. Today, the study of abiotic stress by in vitro experiments is considered perfectly acceptable because it simulates the field environment in which plants are exposed to adverse conditions in a controlled manner. On the other hand, screening of many plant genotypes will not be time-consuming by tissue culture method. And also, results and conclusions based on biochemical characteristics of the explant under stress conditions could be more valuable criteria [7].

Drought stress is the most prevailing abiotic stress limiting plant growth and efficiency. Varieties differ in their sensitivity to extreme environmental factors, so one of the most critical breeding ideas could be selecting and improving the tolerance of plants. One of the most important factors limiting crop efficiency under drought stress is photosynthesis inhibition through the reduction of photosynthetic pigments content [8] and the inhibition of photochemical activity [9]. Afterward Water deficit negatively affects plant hydraulic balance represented by a decrease in relative water content (RWC), stomatal conductivity, and transpiration rate of leaves [10]. Diminished photosynthesis and respiration rate lead to the generation and accumulation of reactive oxygen species (ROS) and subsequently, oxidative damage to cell compartments [11].

Silicon plays a major role in plant growth and development as an essential element [12]. It has been demonstrated that silicon by different methods may enhance plant efficiency and improve plant tolerance to a variety of biotic and abiotic stress [13, 14]. It is assumed that nanoparticles are an alternative tool to overcome different challenges in crop productivity, such as increasing quantitative and qualitative factors of different crops either in stressed or non-stressed conditions with enhancing elements’ efficiency. In fact, plant cells absorb silicon nanoparticles (SiO_2_-NPs), which increases tolerance to stress [15] including extreme temperatures and drought [16–18] by enhancing cell wall rigidity [19]. According to Hajizadeh et al. [20], SiO_2_-NPs could improve the growth and biochemical and physiological traits of *Gerbera jamesonii* under salinity (30 mM) by increasing Ca and K absorption and decreasing Na absorption. The application of SiO_2_-NPs in strawberries exposed to salt [21] and drought [22] showed potential for modulating stresses by increasing antioxidant enzyme activities, such as CAT, APX, GPX, and SOD, and decreasing MDA and H_2_O_2_ content. Avestan et al. [23] suggested that the addition of SiO_2_-NPs to the MS (Murashige and Skoog) medium improved the proliferation and growth of apple explants.

Based on Al-Yasi et al. [24], the Damask rose is a plant with moderate tolerance to drought stress. This species uses two major mechanisms for drought tolerance including osmotic and elastic adjustment in 25% FC. It was shown that the application of SiO_2_-NPs at 50 and 100 mg L^− 1^ concentrations increased the proliferation of apple explants in control plants [25]. Under 15% PEG, the growth parameters, protein and chlorophyll content were decreased in *Phoenix dactylifera* explants, while adding 3.6 mM Si to the growth medium increased all these parameters, as well as CAT and SOD [26]. However, it was reported the amount of proline was decreased by adding Si to the medium [27]. The reduction of water resources along with climate change, is the current challenge of agriculture and will be so dangerous in the future. On the other hand, the positive effects of Si, especially in the form of nanoparticles, have been claimed in recent years [28]. Because of the economic importance of the Damask rose, substances like SiO_2_-NPs should be investigated for their protective abilities and the alleviation of the unfavorable influences of drought stress. Based on the previous studies in vitro culture screening allows the selection drought tolerant plants which there are no information in literatures about the screening of rose plant in tissue culture condition under drought stress. And also, the following objectives were focused on in this research: 1) rapid and precise recognition of the tolerant genotype evaluated 15 days after the use of the osmotic solution and SiO_2_-NPs treatment, while this can take one or 2 years in field investigations; 2) expansion of an in vitro selection approach for drought tolerance in Rosaceae families, which has not been done so far. We selected two Iranian landraces of Damask rose with different characteristics to drought tolerance to achieve these goals.

So, the present study aimed to investigate the response of two *Rosa damascena* genotypes ‘Kashan’ and ‘Maragheh’ by adding SiO_2_-NPs under in vitro culture conditions and to evaluate the potential of SiO_2_-NPs for modulating drought stress by measuring physiological and biochemical traits and we can trust to the technique for evaluation and screening more genotypes and choose better them for planting in the arid and semi-arid areas of Iran, which it has not been reported so far.

## Results

### Physiological traits of damask rose in response to SiO_2_-NPs treatment under in vitro drought stress

#### Leaf relative water content (RWC)

**Fig. 1:**
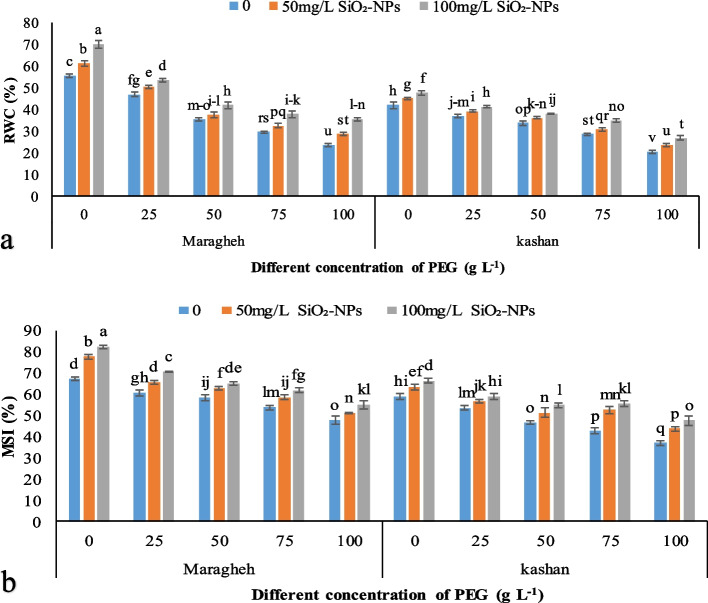
The effect of SiO_2_-NPs application under drought stress induced by PEG on leaf relative water content (RWC) (**a**) and leaf membrane stability index (MSI) of two Damask genotypes (**b**). Different letters indicate significant differences according to the LSD test at *P* < 0.05

Drought stress considerably enhanced RWC content in the Damask rose genotypes and decreased by nanosilicon. The RWC of the genotypes ‘Maragheh’ and ‘Kashan’ subjected to drought stress was reduced by up to 57 and 51%, respectively. However, RWC was increased in the plants treated with SiO_2_-NPs. The highest and the lowest RWC were observed in the rose plants exposed to 100 mg L^− 1^ SiO_2_-NPs × no drought stress and 100 g L^− 1^ PEG × 0 SiO_2_-NPs, respectively. In general, the RWC of genotypes was significantly affected by SiO_2_-NPs, so ‘Maragheh’ had a higher RWC (35.57%) than ‘Kashan’ (26.68%) in severe drought stress × 100 mg L^− 1^ SiO_2_-NPs (Fig. 1a).

#### Membrane stability index (MSI)

In the Damask rose explants subjected to drought stress MSI was reduced, while it was ameliorated by the SiO_2_-NPs application. The maximum and minimum MSI belonged to the genotype ‘Maragheh’ treated with 100 mg L^− 1^ SiO_2_-NPs without drought stress and the genotype ‘Kashan’ treated with 100 g L^− 1^ PEG without any treatment. The reduction of MSI was observed in ‘Kashan’ (37.3%) and ‘Maragheh’ (28.9%) in 100 g L^− 1^ PEG × 100 mg L^− 1^ SiO_2_-NPs compared to the plants subjected to 100 g L^− 1^ PEG × 0 mg L^− 1^ SiO_2_-NPs (Fig. 1b). The diminution of MSI in the genotype ‘Kashan’ was more than the genotype ‘Maregheh’ by 58 and 40% under drought stress and by 24 and 21% in the SiO_2_-NPs treatment, respectively.

#### Photosynthetic pigments

**Fig. 2:**
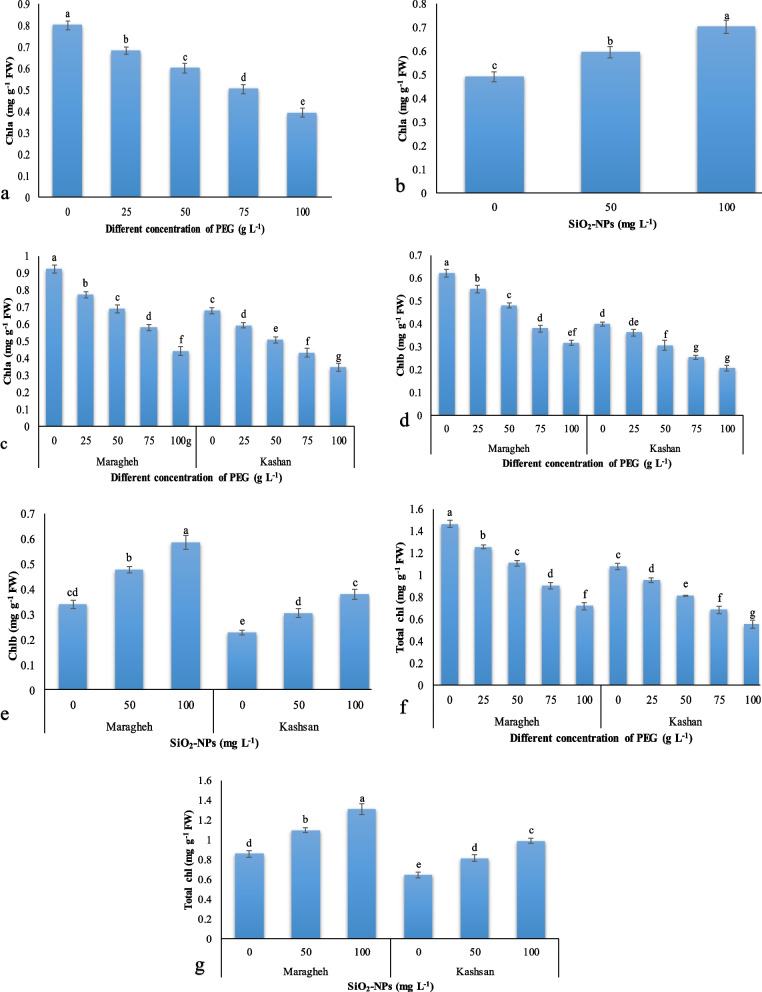
The effect of drought stress on Chl *a* (**a**), SiO_2_-NPs on Chl *a* (**b**), drought stress × genotype on Chl *a* (c), drought stress × genotype on Chl *b* (**d**), SiO_2_-NPs × genotype on Chl *b* (**e**), drought stress × genotype on total Chl (**f**) and SiO_2_-NPs × genotype on total Chl (**g**). Different letters indicate significant differences according to the LSD test at *P* < 0.05

Drought stress decreased Chl *a* by 104% (Fig. 2a), but the application of SiO_2_-NPs improved Chl *a* by 42% (Fig. 2b). Drought stress also reduced Chl *a*, Chl *b*, and total Chl content such that the highest belonged to the control of ‘Maragheh’ without any treatments, and the lowest was observed under 100 g L^− 1^ PEG in ‘Kashan’, while the reduction was greater in ‘Maragheh’ than ‘Kashan’ (Fig. 2c, d, and f). The SiO_2_-NPs treatment enhanced the Chl *b* and total Chl content of both Damask genotypes. The Chl *b* content was increased more in ‘Maragheh’ (up to 71%) than ‘Kashan’ (up to 66%), while the enhancement of total Chl (Fig. 2g) was the same in the two genotypes (up to 52%) (Fig. 2e).

Drought stress decreased the carotenoid content of the genotypes ‘Maragheh’ and ‘Kashan’ by 50 and 58%, respectively, but the SiO_2_-NPs application recovered and increased this trait (Table 1).

**Table 1:** The effect of PEG and SiO_2_-NPs on carotenoid, *Fv*, and *Fv/Fm* contents of the genotypes ‘Maragheh’ and ‘Kashan’

Treatment		‘Maragheh’	‘Kashan’	‘Maragheh’	‘Kashan’	‘Maragheh’	‘Kashan’
PEG (g L^− 1^)	SiO_2_-NPs (mg L^− 1^)	Carotenoid (mg g^− 1^ FW)	Carotenoid (mg g^− 1^ FW)	*Fv*	*Fv*	*Fv/Fm*	*Fv/Fm*
0	0	1.627de	1.373j	3.913ab	3.095jk	0.7823a-c	0.7480 g-k
	50	1.723b	1.550 fg	3.977a	3.286gh	0.7913ab	0.762d-g
	100	1.880a	1.730b	4.004a	3.723 cd	0.7980a	0.7927ab
25	0	1.450i	1.247 l	3.543e	2.816 l	0.7657d-f	0.733j-n
	50	1.595ef	1.351jk	3.841bc	2.981 k	0.7713c-e	0.749 g-j
	100	1.712bc	1.469hi	3.920ab	3.104jk	0.7767b-d	0.7607d-g
50	0	1.348jk	1.108n	3.5ef	2.625 m	0.7507f-i	0.7323 k-n
	50	1.513gh	1.249 l	3.683d	2.744 lm	0.7653d-f	0.7227 l-o
	100	1.662 cd	1.363jk	3.841bc	2.756 l	0.7703c-e	0.7380i-l
75	0	1.182 m	1.008o	3.277gh	2.283op	0.7347i-m	0.7053pq
	50	1.336jk	1.093n	3.478ef	2.495n	0.7477 g-k	0.7183n-p
	100	1.503gh	1.182 m	3.673d	2.695 lm	0.7563e-h	0.7327 k-n
100	0	1.078n	0.8637p	3.143ij	2.169p	0.7217 m-o	0.6957q
	50	1.192 m	0.9943o	3.24hi	2.306o	0.733j-n	0.702q
	100	1.316 k	1.108n	3.381 fg	2.472n	0.7427 h-k	0.7090o-q
S. O. V.							
Drought (a)		**	**	**	**	**	**
Treatment (b)		**	**	**	**	**	**
ab		ns	ns	ns	ns	**	**
Genotype (c)		**	**	**	**	**	**
ac		*	*	**	**	ns	ns
bc		ns	ns	ns	ns	**	**
abc		**	**	**	**	**	**

Different letters indicate significant differences in each trait according to the LSD test at *P* < 0.05. ns, * and ** indicate no significant difference, and significant differences at the 5 and 1% probability levels, respectively. S. O. V. stands for sources of variations

#### Fluorescence parameters

**Fig. 3:**
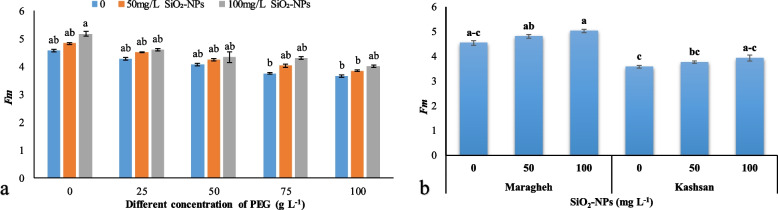
The interactive effect of drought stress × SiO_2_-NPs on *Fm* (**a**) and different levels of SiO_2_-NPs of two Damask genotypes on *Fm* (**b**). Different letters indicate significant differences according to the LSD test at *P* < 0.05

Water deficit had no significant effect on *F*_*0*_ but significantly reduced the *Fm* parameter in the Damask roses. The lowest *Fm* was observed under 75 and 100 g L^− 1^ PEG × 0 mg L^− 1^ SiO_2_-NPs treatment (Fig. 3a), while the plants treated with 100 mg L^− 1^ SiO_2_-NPs had the highest *Fm* (Fig. 3b). Furthermore, water deficit reduced *Fv* and *Fv*/*Fm* in both genotypes (Table 1); Nonetheless, SiO_2_-NPs led to an enhancement in the attributes under drought stress compared to the plants that were not treated with SiO_2_-NPs. However, *Fv* and *Fv*/*Fm* were higher in ‘Maragheh’ either with or without SiO_2_-NPs application than ‘Kashan’ (Table 1).

### Biochemical traits of damask rose in response to SiO_2_-NPs treatment under in vitro drought stress

#### H_2_O_2_ and MDA

According to the results in Table 2, the H_2_O_2_ and MDA contents were increased in both genotypes under drought stress. For example, the highest H_2_O_2_ (3.89 μg L^− 1^) and MDA (4.12 nm g^− 1^ FW) were related to the genotypes ‘Maragheh’ and ‘Kashan’ treated with 100 g L^− 1^ PEG, respectively, while the lowest H_2_O_2_ and MDA contents belonged to ‘Maragheh’ under severe drought stress and the application of 100 mg L^− 1^ SiO_2_-NPs. So, the Damask rose genotypes ‘Maragheh’ and ‘Kashan’ explants supplemented with 100 mg L^− 1^ SiO_2_-NPs revealed lower H_2_O_2_ (30 and 14%) and MDA (48 and 24%) contents (Table 2) under 100 g L^− 1^ PEG, respectively.

**Table 2:** The effect of PEG and SiO_2_-NPs on H_2_O_2_, MDA, and protein content of the genotypes ‘Maragheh’ and ‘Kashan’

Treatment		‘Maragheh’	‘Kashan’	‘Maragheh’	‘Kashan’	‘Maragheh’	‘Kashan’
PEG (g L^−1^)	SiO_2_-NPs (mg L^− 1^)	H_2_O_2_ (μg L^− 1^)	H_2_O_2_ (μg L^− 1^)	MDA (nmol g^− 1^ FW)	MDA (nmol g^− 1^ FW)	Protein (mg g^− 1^ FW)	Protein (mg g^− 1^ FW)
0	0	1.76o	1.647p	1.315q	1.765no	1.95d	1.8ef
	50	1.403r	1.511q	1.021r	1.255q	2.017c	1.951d
	100	1.203 s	1.274 s	0.8767r	0.8823r	2.209a	2.116b
25	0	2.371 l	2.61ij	2.484hi	2.639gh	1.807ef	1.446jk
	50	2.268 m	2.465 k	2.098kl	2.269jk	1.417kl	1.495j
	100	2.163n	2.308 lm	1.508p	1.864 m-o	1.647hi	1.656 h
50	0	2.919f	2.921f	2.780 fg	3.015de	1.731 g	1.251n
	50	2.542jk	2.683hi	2.355ij	2.779 fg	1.842e	1.327 m
	100	2.279 m	2.464 k	1.714o	2.481hi	1.927d	1.436 k
75	0	3.071de	3.123d	3.039c-e	3.217c	1.603i	0.9903q
	50	2.746gh	2.826 g	2.52hi	2.893ef	1.645hi	1.17o
	100	2.606ij	2.585j	1.9mn	2.574 h	1.797ef	1.231n
100	0	3.893a	3.518b	3.817b	4.122a	1.381 l	0.791 s
	50	3ef	3.215c	2.775 fg	3.78b	1.626hi	0.8983r
	100	2.721 h	3.012e	1.988 lm	3.12 cd	1.759 fg	1.074p
S. O. V.							
Drought (a)		**	**	**	**	**	**
Treatment (b)		**	**	**	**	**	**
ab		**	**	**	**	**	**
Genotype (c)		**	**	**	**	**	**
ac		**	**	**	**	**	**
bc		**	**	**	**	**	**
abc		**	**	**	**	**	**

Different letters indicate significant differences in each trait according to the LSD test at *P* < 0.05. ns, * and ** indicate no significant and significant differences at the 5 and 1% probability levels, respectively. S. O. V. stands for sources of variations

#### Total soluble protein content (TSP)

The TSP content showed a significant difference between the two genotypes exposed to the SiO_2_-NPs treatment under drought stress. According to Table 2, protein content had a decreasing trend in both genotypes along with increasing the PEG concentration. The ‘Maragheh’ Damask explants treated with 100 mg L^− 1^ SiO_2_-NPs had the highest TSP content (2.2 mg g^− 1^ FW) and the ‘Kashan’ Damask explants subjected to 100 g L^− 1^ PEG without SiO_2_-NPs had the lowest content of protein (0.7 mg g^− 1^ FW). With increasing the PEG concentration, the TSP content was diminished by 41 and 89% in ‘Maragheh’ and ‘Kashan’, respectively. However, the rose explants treated with 50 and 100 mg L^− 1^ SiO_2_-NPs slowed down the reduction process by 19 and 20% in ‘Maragheh’ and 56 and 49% in ‘Kashan’, respectively (Table 2). In general, ‘Maragheh’ under severe drought stress had a higher TSP content than ‘Kashan’.

#### Proline

**Fig. 4:**
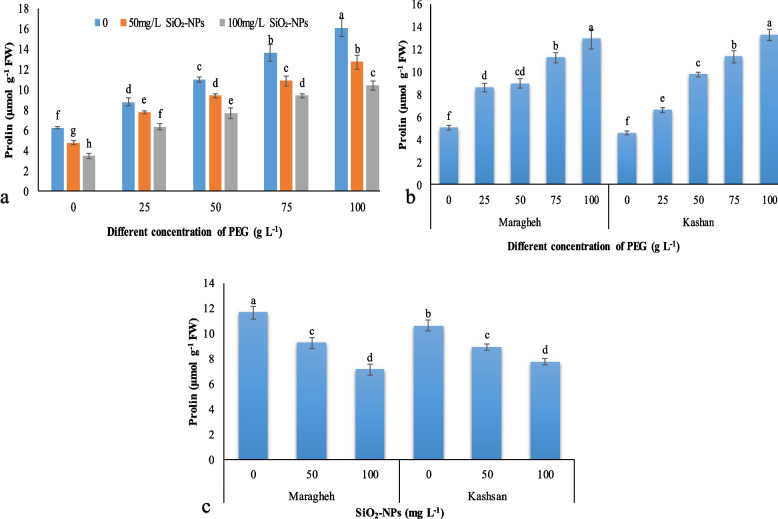
The interactive effect of drought stress × SiO_2_-NPs on leaf proline content (**a**), drought stress × genotype on proline content (**b**), and SiO_2_-NPs × genotype on proline content. Different letters indicate significant differences according to the LSD test at *P* < 0.05

The PEG-induced drought stress and SiO_2_-NPs treatment of the Damask roses were caused to significant differences in proline content (Fig. 4a). Increasing drought stress led to an enhancement of proline content which 100 g L^− 1^ PEG enhanced it 2.5 folds than the control plants. However, the explants subjected to SiO_2_-NPs revealed a reduction in proline content, and 100 mg L^− 1^ SiO_2_-NPs was more effective than 50 mg L^− 1^ SiO_2_-NPs that declined 21 and 35%, respectively at higher PEG treatment compared to the control (Fig. 4a). The results showed that proline content increased in both Damask rose genotypes under PEG-induced drought stress (Fig. 4b). The effect of SiO_2_-NPs in the two Damask rose genotypes revealed that proline content was higher in ‘Maragheh’ by 9% than ‘Kashan’ (Fig. 4c), and there was no significant difference in other concentrations of SiO_2_-NPs between the two genotypes as illustrated in Fig. 4c.

#### Antioxidative enzyme activity

With increasing the PEG concentration, the GPX and POD activities were enhanced in both genotypes (Table 3) although ‘Maragheh’ had higher GPX activity in 100 g L^− 1^ PEG than ‘Kashan’. However, the Damask rose explants exposed to different levels of SiO_2_-NPs up-regulated the POD and GPX activities. According to Table 3, the effect of SiO_2_-NPs, especially at the high level (100 mg L^− 1^), was very obvious in modulating the activities of these enzymes in ‘Maragheh’. Also, increasing the concentration of PEG and SiO_2_-NPs increased SOD and CAT activities (Table 3).

**Table 3:** The effect of PEG and SiO_2_-NPs on the SOD, POD, GPX, and CAT activity of the genotypes ‘Maragheh’ and ‘Kashan’

Treatment		‘Maragheh’	‘Kashan’	‘Maragheh’	‘Kashan’	‘Maragheh’	‘Kashan’	‘Maragheh’	‘Kashan’
PEG (g L^−1^)	SiO_2_-NPs (mg L^− 1^)	SOD (Unit mg^− 1^ protein)	SOD (Unit mg^− 1^ protein)	POD (Unit mg^− 1^ protein)	POD (Unit mg^− 1^ protein)	GPX (Unit mg^− 1^ protein)	GPX (Unit mg^− 1^ protein)	CAT (Unit mg^− 1^ protein)	CAT (Unit mg^− 1^ protein)
0	0	0.4154u	0.1762y	0.01323n	0.01283n	0.223 t	0.1681u	0.1171q	0.1926o
	50	0.498 t	0.2552x	0.01537mn	0.0149mn	0.3114r	0.2693 s	0.1575p	0.2010o
	100	0.525 s	0.3344w	0.01907 l-n	0.0231 k-n	0.3634op	0.3087r	0.1965o	0.2205n
25	0	0.5152 s	0.3517v	0.0238j-n	0.02553j-n	0.3765no	0.3446q	0.1966o	0.2259n
	50	0.567r	0.4168u	0.02863i-n	0.02873i-n	0.4187 l	0.3965 m	0.2491 m	0.2629 m
	100	0.6696p	0.4832 t	0.0333 h-l	0.0338 h-l	0.5044i	0.4524jk	0.3652i	0.2884 l
50	0	0.8178 l	0.5591r	0.02967i-m	0.03383 h-l	0.4967i	0.3586pq	0.3452j	0.258 m
	50	0.9485 h	0.602q	0.0357 g-k	0.03957f-j	0.538 h	0.4384 k	0.3877 h	0.3637i
	100	1.032e	0.7318o	0.04643d-h	0.04607e-h	0.6156f	0.5842 g	0.4536f	0.416 g
75	0	0.8704 k	0.7557n	0.03737f-k	0.04783c-h	0.5928 g	0.3842mn	0.4293 g	0.3087 k
	50	1.007f	0.7898 m	0.0519c-g	0.0527c-f	0.675e	0.5418 h	0.4893e	0.3796hi
	100	1.169b	0.9131j	0.0632bc	0.05927b-e	0.761c	0.7583c	0.5555c	0.4667f
100	0	0.9298i	0.903j	0.0432e-i	0.05927b-e	0.7097d	0.4655j	0.5353d	0.3671i
	50	1.115d	0.9706 g	0.0626b-d	0.06317bc	0.7833b	0.5934 g	0.5926b	0.4207 g
	100	1.305a	1.153c	0.08167a	0.0704ab	0.885a	0.7978b	0.6802a	0.5205d
S. O. V.									
Drought (a)		**	**	**	**	**	**	**	**
Treatment (b)		**	**	**	**	**	**	**	**
ab		**	**	**	**	**	**	**	**
Genotype (c)		**	**	**	**	**	**	**	**
ac		**	**	ns	ns	**	**	**	**
bc		**	**	**	**	**	**	**	**
abc		**	**	**	**	**	**	**	**

Different letters indicate significant differences in each trait according to the LSD test at *P* < 0.05. ns, * and ** indicate no significant and significant differences at the 5 and 1% probability levels, respectively. S. O. V. stands for sources of variations

On the other hand, the genotype ‘Maragheh’ had higher enzyme activity than ‘Kashan’ so the SOD activity was 1.30 and 1.15 Unit mg^− 1^ protein and the CAT activity was 0.68 and 0.52 Unit mg^− 1^ protein under 100 g L^− 1^ PFG × 100 mg L^− 1^ SiO_2_-NPs in ‘Maragheh’ and ‘Kashan’, respectively. The application of 100 mg L^− 1^ SiO_2_-NPs × severe drought stress up-regulated the SOD activity by 41 and 28% in ‘Maragheh’ and ‘Kashan’, respectively, and 100 mg L^− 1^ SiO_2_-NPs was more effective than 50 mg L^− 1^ SiO_2_-NPs while the CAT activity was increased by up to 28 and 44% in ‘Maragheh’ and ‘Kashan’ at the same conditions, respectively (Table 3).

### Multivariate analysis of damask rose genotypes under PEG and SiO_2_-NPs treatments

**Fig. 5:**
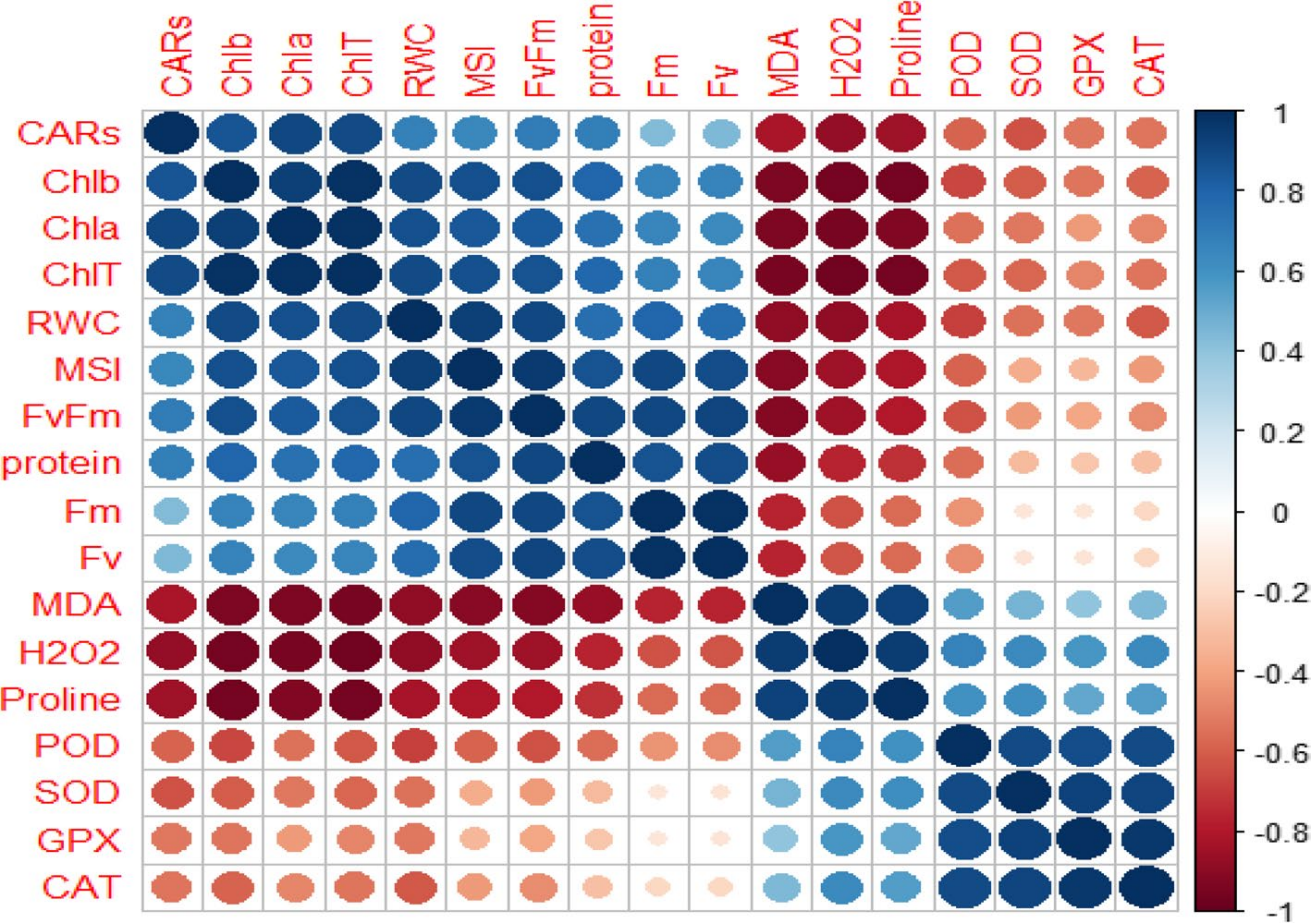
Pearson correlation analysis of SiO_2_-NPs treatment and variable trait relationship in damask under control and different drought conditions. The heat map of the Pearson correlation coefficient (r) values of variable traits, where the colored scale indicates the positive (blue) or negative (red) correlation and the ‘r’ coefficient values (r = − 1.0 to 1.0). The tested variables included carotenoids (CARs), Chl *a*, Chl *b*, ChlT, relative water content (RWC), membrane stability index (MSI), maximum PSII (*Fv/Fm*), protein (Pro), maximal fluorescence from dark-adapted leaf (*Fm*), variable fluorescence (*Fv*), malondialdehyde (MDA), hydrogen peroxidase (H_2_O_2_), proline (Pro), peroxidase (POD), superoxide dismutase (SOD), guaiacol peroxidase (GPX), and catalase (CAT)

The analysis of Pearson correlation demonstrated that the photosynthetic pigments were positively correlated with RWC and MSI, chlorophyll fluorescence parameter, and protein. Similarly, the correlations detected between MAD, proline, and H_2_O_2_ were positive while they displayed a negative correlation with photosynthetic pigments, RWC, chlorophyll fluorescence parameters, and protein. The antioxidant enzymes, including SOD, POD, GPX, and CAT, had a significant positive correlation with each other, which is illustrated in Fig. 5.

**Fig. 6:**
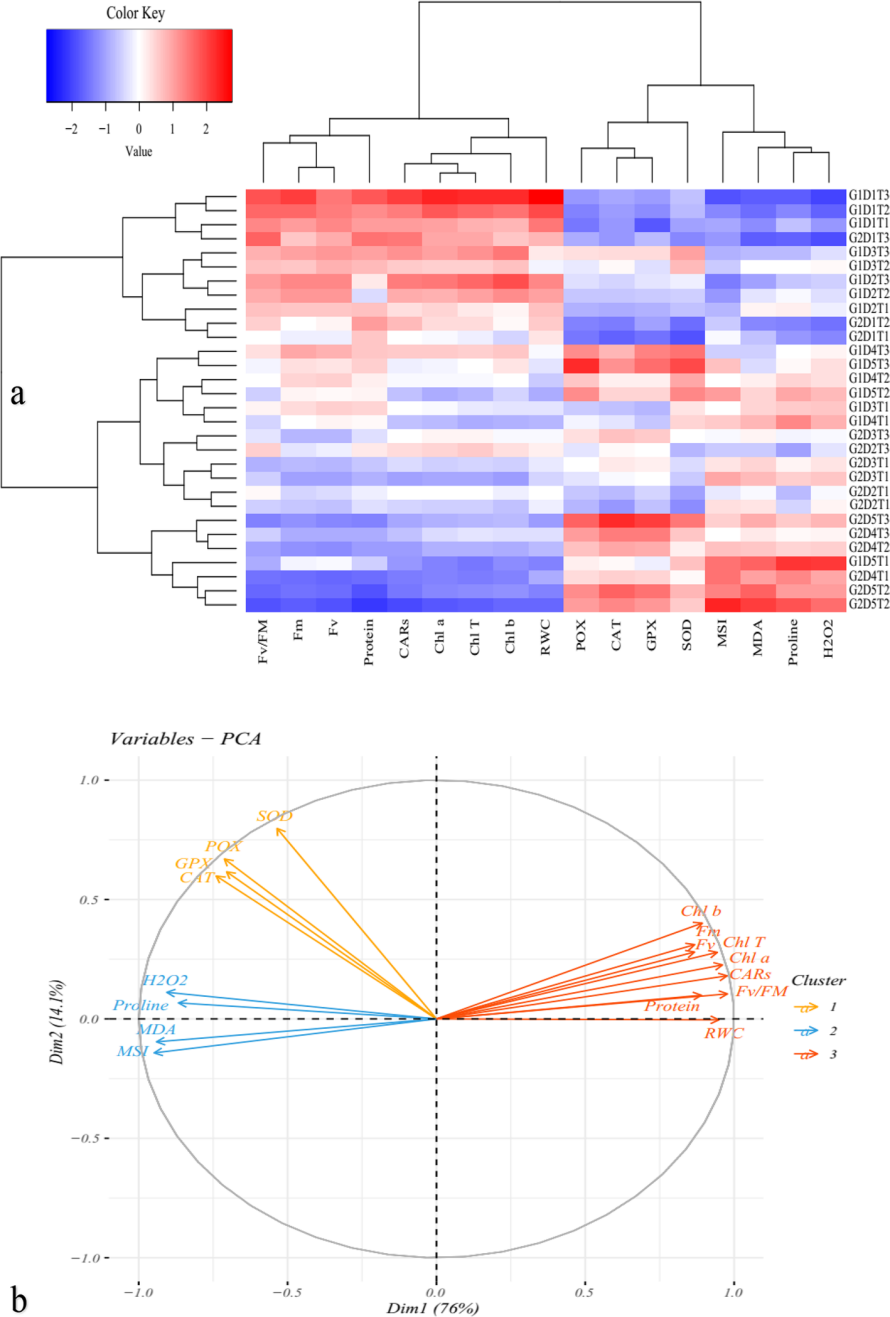
The heat map (**a**) and loading biplot of the recorded traits (**b**) of the physiological and biochemical alterations in *Rosa damascena* genotypes under induced-drought stress by PEG and treated with SiO_2_-NPs application under in vitro culture. The heat map represents relative water content (RWC), membrane stability index (MSI), chlorophyll *a* (Chl *a*), chlorophyll *b* (Chl *b*), total chlorophyll (Total Chl), carotenoids (CARs), proline content, malondialdehyde (MDA), H_2_O_2_ content_,_ total soluble protein content, guaiacol peroxidase (GPX) activity, catalase (CAT) activity, superoxide dismutase (SOD) activity, *Fv*, *Fm* and *Fv/Fm*. G, D, and T for Damask rose genotypes, drought stress induced by PEG, and SiO_2_-NPs

The Heat map analysis based on the reaction of the Damask rose genotypes to the SiO_2_-NPs treatment under water deficit induced by PEG applications in in vitro conditions uncovered that the attributes including proline, H_2_O_2_, MDA, MSI, POD, GPX, CAT, and SOD activity had a positive correlation with drought stress although the SiO_2_-NPs treatment decreased them at moderate water deficit. On the other hand, some traits, such as RWC, Chl *a*, *b* and total, CARs, total soluble protein content, *Fv*, *Fm*, and *Fv/Fm*, showed a negative correlation with drought stress, but the SiO_2_-NPs application modulated the traits (Fig. 6a).

Cluster analysis and dendrograms in the heat map (Fig. 6) showed three major groups in the measured traits of the Damask rose genotypes under drought stress and SiO_2_-NPs application. Group I contained RWC, photosynthesis pigments, total soluble protein content, *Fv*, *Fm*, and *Fv/Fm*; group II contained antioxidant enzyme activity, and group III contained MSI, MDA, proline, and H_2_O_2_ content (Fig. 6a). Moreover, the biplot of the variables confirmed the heat map cluster analysis in which the traits were classified into three groups as already mentioned (Fig. 6b). In general, the cluster analysis of the heat maps for the Damask rose genotypes treated with SiO_2_-NPs under drought stress induced by PEG treatments indicated two main groups. Group I included the Damask rose of ‘Maragheh’ treated with 0, 50, and 100 mg L^− 1^ of SiO_2_-NPs under drought stress induced with 0, 25, and 50 g L^− 1^ PEG and Damask rose ‘Kashan’ treated with 0, 50, and 100 mg L^− 1^ of SiO_2_-NPs in the absence of drought stress. Group II included ‘Maragheh’ under severe drought stress (75 and 100 g L^− 1^ of PEG) and treated with 0, 50, and 100 mg L^− 1^ of SiO_2_-NPs and ‘Kashan’ under zero, moderate, and severe drought stress and treated with 0, 50 and 100 mg L^− 1^ of SiO_2_-NPs (Fig. 6a).

## Discussion

### Physiological traits

One of the most suitable traits for measuring the plant hydraulic balance in water deficiency is leaf relative water content (RWC). According to the results, the water status of ‘Maragheh’ was higher than ‘Kashan’, especially under severe drought stress. The application of 100 mg L^− 1^ SiO_2_-NPs induced 51 and 29% increases in RWC under severe drought stress in ‘Maragheh’ and ‘Kashan’, respectively. The same findings were demonstrated by Hajizadeh et al. [20] and Ahmadian et al. [29] under salinity and drought stress, respectively. The accumulation of silicon in the cell wall apoplast of the leaf tissue led to higher strength [30]. In this regard, it has been demonstrated that sedimentation of silicon in the endodermal walls of cells, as an apoplastic fluid, keeps moisture of the plant under water deficiency [31]. Under drought stress, lipid peroxidation decreases cell membrane stability. On the other hand, increasing membrane stability index by silicon treatment has also been reported in various studies [32, 33]. The genotype ‘Maragheh’ treated with 100 mg L^− 1^ SiO_2_-NPs without drought stress had a stronger membrane with a high membrane stability index (82.28%), while the lowest MSI was related to ‘Kashan’ (36.91%) exposed to 100 g L^− 1^ PEG without SiO_2_-NPs. This result is complemented by the MDA content, which was the highest in ‘Kashan’ under severe water deficit without SiO_2_-NPs. Silicon can decrease the adverse effects of water deficiency via an increase in water uptake and/or the decrease in transpiration, modulation in the cell wall formation, increase the strength of individual organelles of the plant [34], finally enhances photosynthesis and improves the plant tolerance to drought stress [35]. Drought stress significantly reduced the content of photosynthetic parameters in water deficit-stressed plants versus the control, especially for the 100 g L^− 1^ PEG treatment where there was a 38% decrease in the total chlorophyll. The carotenoid content also showed the highest decline in ‘Kashan’ under severe drought stress and without SiO_2_-NPs treatment. The reduction of chlorophyll biosynthesis under water deficit stress can be related to the competition between glutamyl kinase (a catalyzing enzyme of proline) [36] and glutamate ligase (the first enzyme in the biosynthetic pathway of chlorophyll) [37], which caused glutamate precursors to be used more for proline biosynthesis than for chlorophyll biosynthesis. Also, the up-regulation of the chlorophyllase activity under drought stress can be the other reason for the loss of chlorophyll [38]. The treatment with SiO_2_-NPs increased Chl *a*, Chl *b*, and total chlorophyll and carotenoid content in comparison with the plants under stress and non-stress conditions and without SiO_2_-NP. The beneficial effects of Si or SiO_2_-NPs in water-stressed plants could be ascribed to the increased photosynthetic efficiency, and stomatal conductivity using increased potassium uptake, which is responsible for stomatal conductivity and translocation of potassium to the guard cells of stomata [39], and water use efficiency; traits that, in turn, improved plant tolerance [40]. The same results regarding the improvement of photosynthetic efficiency rate were observed in our study. However, the carotenoid content of the leaves was increased in severe drought stress by 18 and 22% in ‘Maragheh’ and ‘Kashan’, respectively as treated with 100 mg L^− 1^ SiO_2_-NPs. These findings were in agreement with Ghorbanpour et al. [41] results.

The reduction of the *Fv/Fm* values reveals serious damage to PSII and possible changes in plant photosynthetic rate exposed to stress conditions [42]. In the present work, fluorescence chlorophyll parameters decreased significantly under drought stress; however, the treatment with SiO_2_-NPs, especially at the 100 mg L^− 1^ concentration, decreased them at a lower rate. With the increase in drought stress from 0 to 100 g L^− 1^, the maximum PSII efficiency was decreased by 7.6 and 6.7% in ‘Maragheh’ and ‘Kashan’, respectively. However, the application of 100 mg L^− 1^ SiO_2_-NPs led to an increase in the efficiency of photosynthesis so that ‘Maragheh’ had higher photosynthetic efficiency than ‘Kashan’. Lower *Fv*/*Fm* in this study can be related to the damage to chloroplasts, which can be verified with the data related to chlorophyll reduction. Earlier work has shown that silicone increases photosynthetic pigments in various plants in stressed and control conditions [43]. In this work, although drought stress was related to lower *Fv*/*Fm* value, they were significantly higher in Damask explants treated with Si under drought stress. The findings indicated that drought stress led to a decrease in *Fm* and *Fv* values. Probably the reason for the positive effect of Si in maintaining plant hydraulic balance and increasing stomatal conductivity against more water loss is higher water uptake as demonstrated by Shen et al. [35]. Silicon transmits light to the leaf mesophyll, which is the photosynthetic active center and increases the photosynthesis rate [44] by increasing *Fv*/*Fm* values [45], improving the maximum performance of Quantum PSII and preserving the integrity of chloroplasts despite the severe oxidative stress [46]. Moreover, it has been demonstrated that Si most likely has a cofactor role in most enzymatic reactions involved in mesophyll biosynthetic pathways [47]. Therefore, the Si-treated explants preserved a higher amount of chlorophyll under drought stress conditions, which is consistent with Maghsoudi et al. [44]. According to the results of Atal et al. [48], a decrease in *Fm* was also observed. Kaufman et al. [49] suggested that silicon settled in the epiderm of plant cells in the form of silica and improved photosynthetic efficiency by transferring light to the mesophyll as mentioned before.

### Biochemical traits

Water deficit is closely attributed to the production of ROS, especially hydrogen peroxide and superoxide anion in water deficit conditions, which may, in turn, damage membranes and cause electrolyte leakage [50]. In the present study, against the increased levels of CAT and ascorbate peroxidase activities, higher levels of H_2_O_2_ and MDA were accumulated in drought-stressed explants, which might be due to enhanced photorespiration. However, the application of SiO_2_-NPs decreased the amount of H_2_O_2_ and MDA in the genotype ‘Maragheh’ more than ‘Kashan’. Similar to our results, Gunes et al. [51], Shi et al.Also demonstrated that the amount of MDA decreased in Si-treated sunflowers during water deficit stress. [52]silicon caused to increase in the activity of SOD and CAT and improved water uptake by tomatos observed that .

Proline plays a role as an osmoprotectant molecule and is accumulated under water deficit and salinity [53], which is observed in the present work, as well. In our study, leaf proline concentration was significantly increased in drought-stressed plants but not in response to the application of nano silicon, which was not in line with some other researchers [54] although some reports have shown a reduction in proline content with increasing the concentration of applied silicon [55]. According to the interaction between drought and SiO_2_-NPs, the lowest amount of proline was related to the control plants treated with 100 mg ^−^ L^1^ SiO_2_-NPs. In some plants, changes in proline levels are related to their stress tolerance [56] as proline causes the hydration of biomolecules and serves as energy as a nitrogen reserve source [57]in some of the studies tolerant than ‘Kashan’ . ‘Maragheh’ with a high level of proline and protein content seems to be more water stress-[58]. The plant protective systems for overcoming the harmful effects of ROS derived from water deficit stress use antioxidant enzymes including SOD, glutathione peroxidase, and CAT and in this regard, Cu/Zn-SOD, is more important than other antioxidant enzymes under water deficit [59]. It seems that the ability of each enzyme in scavenging free radicals differs from species to species as the activity of antioxidant enzymes, such as GPX, POD, CAT, and SOD, in ‘Maragheh’ were higher than ‘Kashan’ and also the application of SiO_2_-NPs up-regulated them in ‘Maragheh’. Saed-Moucheshi et al. [60] demonstrated that plants with high levels of antioxidant enzyme activities are more resistant to oxidative damage as the activity of component enzymes are usually only doubled in response to many stress conditions. According to earlier studies, the application of exogenous silicon improves the ROS scavenging capability of antioxidant enzymes by regulating their activities [61, 62]. Similar to Gong et al. [63], the treatment of wheat plants with silicon led to high drought tolerance by increasing the activities of antioxidant enzymes, including CAT, SOD, and glutathione reductase. Also, Shi et al. [52] reported an increase in the activities of SOD and CAT in Si-treated tomato plants under water deficit. The mechanisms of the silicon in increasing the activities of the antioxidative enzymes can be related to the protection of cell membranes via the prevention of proteases from access to the internal proteins of the membrane and also preventing membrane disruption and loss of integrity [63].

## Conclusion

The present work aimed to investigate the physiological and biochemical responses of two Damask rose ‘Maragheh’ and ‘Kashan’ to different water deficits and compare their tolerance in response to SiO_2_-NPs. Water deficiency leads to adverse effects in plants, which is associated with the reduction of photosynthetic pigments and reduceed the performance, but the Damask rose ‘Maragheh’ uses the mechanism of osmotic regulation by increasing the amount of proline, protein and antioxidative enzymes activities to withstand against drought stress.

According to the modulative effects of SiO_2_-NPs under drought stress which are more obvious in preserving the strength and of leaf structure, and also its key role in biochemical processes, including the intracellular synthesis of organic compounds, it seems that treatment of Damask roes with SiO_2_-NPs under drought stress is an appropriate way for the cultivation of Damask rose in arid and semi-arid origins of Iran to have an economical performance. According to the positive effects of SiO_2_-NPs in both genotypes under control conditions in proline production, antioxidative enzyme activation, and photosynthetic pigments preservation, So, the use of genotypes with inherent tolerance potential to water deficit, will double the SiO_2_-NPs efficiency in achieving this goal. These results suggested that ‘Maragheh’ may tolerate water deficiency better than ‘Kashan’ by treating it with SiO_2_-NPs.

## Methods

### Experiment design

**Fig. 7:**
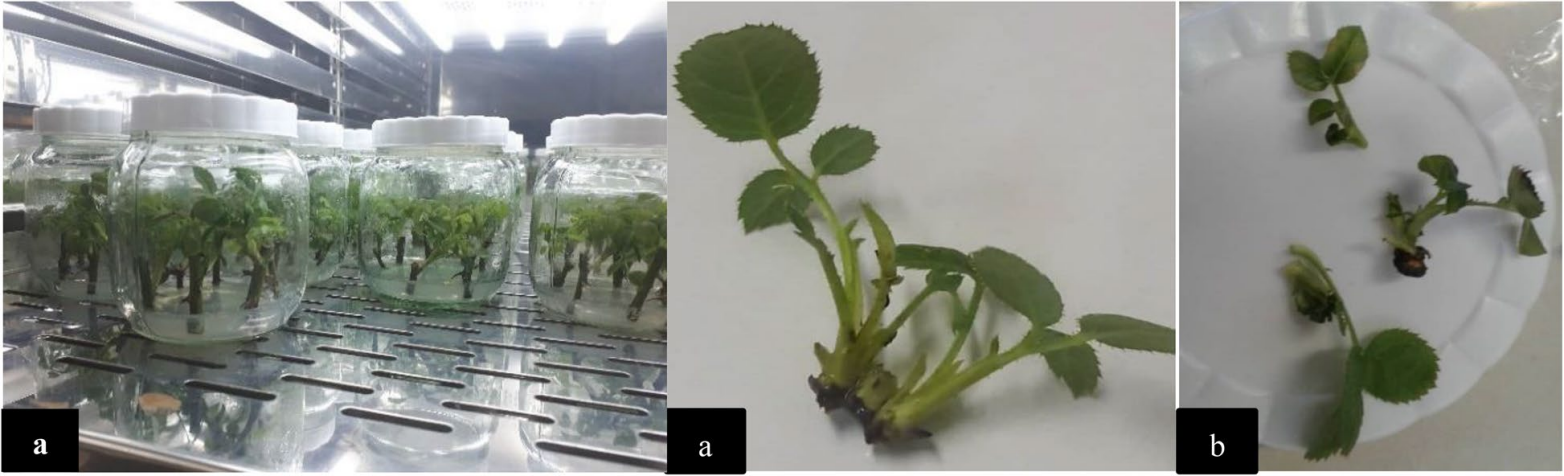
**a** *In vitro* shoot proliferation of Damask rose, **b** the shoots regenerated from one explant.

The purpose of this work was to stimulate drought stress using PEG 6000 and modulate it by SiO_2_-NPs in two Iranian Damask rose genotypes under in vitro conditions. In this case, according to our preliminary experiments about the response of different genotypes to water deficiency, two genotypes of Damask rose were chosen for the present work [64]. The research was conducted as a factorial experiment based on a completely randomized design with three factors. The first factor was two genotypes (‘Maragheh’ and ‘Kashan’), the second factor was PEG concentration (0, 25, 50, 75, and 100 g L^− 1^) and finally, the third factor was SiO_2_-NPs concentration (0, 50, and 100 mg L^− 1^) with three replications. Explants were selected from one-year branches (0.4-0.6 cm in diameter) of two local Damask roses (*R. damascena* Mill.) from the University of Maragheh in northwest of Iran (37.3892° N, 46.2534° E) and Kashan (33.9850° N, 51.4100° E) in the central region of Iran. The plant material and shoots for wild collections were obtained under the supervision and permission of the Maragheh University guidelines and according to national guidelines and all authors complied with all the local and national guidelines. The central part of the vegetative shoots of three-year-old Damask roses at the active growth stage having axillary buds were chosen for the experiment. At the first, 1.5-2 cm of the shoot explants were disinfected with 10% (v/v) NaOCl (5.25%) for 20 min and then rinsed with running tap water for 15 min. The explants were sterilized with 10% Clorox solution for 15 min and then washed three times in ddH_2_O. Finally, they were planted in culture bottles having 25 ml of MS medium [65] and vitamins plus 7.5 g L^− 1^ Agar combined with minerals and 30 g L^− 1^ sucrose. The pH of the media was adjusted to 5.7 using NaOH or HCl. All culture vessels containing explants were placed in a growth chamber at 25 °C and 8 hours of darkness, 16 hours of lightness, and 60-70% humidity. Approximately 7 days after the establishment of the explants, the first traces of bud growth appeared, and finally, after four to 5 weeks, when the explants had grown sufficiently, they were taken out of the growth chamber to be filled and placed in a proliferating MS medium including 360 μg^− 1^ L BA and 30 μg L^− 1^ NAA. For the experiment we used proliferated plants (~ 4 cm) after 35 days as an experiment explant (Fig. 7) and transferred them to sterile culture vessels including 25 ml of MS medium (five shoots per culture vessel) as experiment materials.

### Preparing the treatment medium including PEG and SiO_2_-NPs

Polyethylene glycol was used to induce drought stress. For this purpose, the treatments were applied at five levels (0, 25, 50, 75, and 100 g L^− 1^) or with an osmotic pressure of 0, − 0.2, − 0.5, − 0.7, and − 0.9 MPa on two genotypes. After preparing the propagation medium, the shoots were placed in the culture medium. After preparing the concentrations and complete dissolution of PEG in water and adjusting the pH, it was added to the culture medium so that it was one centimeter higher than the medium, then five shoots were placed in each bottle and transferred to the growth chamber and the level of proliferation of explants was evaluated after 4 weeks. The nanoparticles of silicon (size<50 nm) used in our experiment were bought from NANOSANY Corporation (Mashhad, Iran) the same as our last work [20], and prepared at three levels (0, 50, and 100 mg L^− 1^). They were, then, supplemented to the culture medium in phase suspension in MS medium [66]. Then, five shoots were placed in each glass and transferred to the growth chamber. After about 14 days, there were collected and their traits were measured. All in vitro cultures were maintained at 23 ± 2 °C under a 16/8 h day/night photoperiod provided by cool white fluorescent lamps at 40 μmol m^− 2^ s^− 1^ (Philips TLD 36 W/95). After about 14 days, they were collected to measure the traits.

### Measurement of physiological traits of *Rosa damascena*

#### Leaf relative water content

The amount of leaf RWC was determined in the fully expanded topmost leaf of the explants. At first, the fresh weight of the leaves was recorded and then they were plunged in ddH_2_O in a Petri dish. After 2 hours and removing the surface water of the samples, their turgid weights were recorded. The sample leaves were then placed in an oven at 70 °C and dried to reach a stable weight. The leaf RWC was calculated with the method described by Turner [67] as the following formula (1):

1 $${\displaystyle \begin{array}{c}\textrm{RWC}\%=\left(\textrm{Fresh}\ \textrm{Weight}-\textrm{Dry}\ \textrm{Weight}\right)\times 100\\ {}\textrm{Turgid}\ \textrm{Weight}-\textrm{Dry}\ \textrm{Weight}\end{array}}$$

#### Membrane stability index

The leaves were cut into small samples of the same size. Then, the leaf discs were weighed and transferred to the test tubes containing 10 mL of ddH_2_O. The tubes were transferred to a water bath at 40 °C for 30 min and then the EC of the samples was recorded. The samples were placed in other test tubes and incubated at 100 °C in the boiling water bath for 15 min, and their EC was recorded as mentioned before. The amount of MSI was evaluated by the following formula (2) [68]:

2 $$\textrm{EL}\%=\left[\ \textrm{EC}1/\textrm{EC}2\right)\Big]\times 100$$

#### Measurement of photosynthetic pigments and chlorophyll fluorescence of leaf

Chlorophyll *a*, chlorophyll *b*, total chlorophyll, and carotenoids were evaluated in the leaves of the explants according to Arnon [69] method using a spectrophotometer (Shimadzu, Model UV 1800, Kyoto, Japan) at 470, 663, and 645 nm, respectively and expressed in mg g^− 1^ FW using formula (3)-(6). The chlorophyll parameters of the *Rosa damascena* explants were measured using a portable photosynthesis meter (Walz GmbH Eichenring, 691,090 Efeltrich, Germany) at the end of the experiment. Minimal fluorescence, *F*_*0*_, was evaluated in leaves after 30 min of dark-incubation. Then, these leaf samples were used under full light conditions to determine the maximal fluorescence, *Fm*. Maximal variable fluorescence (*Fv*) and photochemical efficiency of PSII (*Fv/Fm*) were, then, evaluated from the recorded parameters [66].

3 $$\textrm{Ch}1\ a\ \left(\textrm{mg}\ {\textrm{g}}^{-1}\ \textrm{FW}\right)=\left[\right(12.7\left({\textrm{A}}_{663}\right)-\left(2.69\left({\textrm{A}}_{645}\right)\right)\Big]\times \left(\frac{v}{1000w}\right)$$

4 $$\textrm{Chl}\ b\ \left(\textrm{mg}\ {\textrm{g}}^{-1}\textrm{FW}\right)=\Big[\left(22.9\left({\textrm{A}}_{645}\right)-\left(4.68\left({\textrm{A}}_{663}\right)\right)\right]\times \left(\frac{v}{1000w}\right)$$

5 $$\textrm{Total}\ \textrm{Chl}\ \left(\textrm{mg}\ {\textrm{g}}^{-1}\textrm{FW}\right)=\left[\right(20.2\left({\textrm{A}}_{645}\right)+\left(8.02\left({\textrm{A}}_{663}\right)\right)\Big]\times \left(\frac{v}{1000w}\right)$$

6 $$\frac{\textrm{Carotenoids}\ \textrm{content}=\left[100\left({\textrm{A}}_{470}\right)+3.27\left(\textrm{mg}\ \textrm{Chl}\ a\right)-104\left(\textrm{mg}\ \textrm{Chl}\ b\right)\right]/227}{v:\textrm{final}\ \textrm{volume}\ \textrm{and}\ w:\textrm{shoot}\ \textrm{fresh}\ \textrm{weight}}$$

### Measurement of biochemical traits of *Rosa damascena*

#### Hydrogen peroxide (H_2_O_2_) determination

The amount of hydrogen peroxide in the explants was determined with the method previously established by Liu et al. [70]. In this case, 0.5 g of leaf tissues were ground in liquid nitrogen and potassium phosphate buffer (KPB) (pH 6.8). The grounded leaf samples were centrifuged at 7000 rpm for 25 min at 4 °C. A 100-μL aliquot of the supernatant was added to 1 mL of Xylenol solution. The solution was then completely mixed and left to rest for 30 min. The amount of hydrogen peroxide, which is directly related to the intensity of the color and represents its amount in the samples, was evaluated by a spectrophotometer (Shimadzu, Japan) at 560 nm and recorded as μmol g^− 1^ FW.

#### Malondialdehyde (MDA) determination

MDA was determined as 2-thiobarbituric acid (TBA) reactive metabolites [71]. About 1.5 mL of the extract of each sample was homogenized in 2.5 mL of 5% TBA made in 5% Trichloroacetic acid (TCA). The solution was warmed at 95 °C for 15 min and then cooled on ice quickly. After centrifugation at 5000 rpm for 10 min, the amount of the supernatant absorbance was recorded at 532 nm. The level of MDA was measured as nmol g^− 1^FW according to the following equation. (7).

7 $$\textrm{MDA}=1000\times \left[\left(532\textrm{nm}-600\textrm{nm}\right)\times 1.049\right]/155$$

#### Protein determination

The amount of protein was measured following the Bradford method [72], which was calibrated for each determination with the standard bovine serum albumin curve. In this case, 100 mg of the treated explants were placed in a test tube with 2 mL of 50 mM potassium phosphate buffer at pH 7.0. The solution was centrifuged at 7000-12000 rpm. Then, the supernatant was recovered and centrifuged at 3000 rpm for 15 min at 4 °C. The samples were prepared with 1:100 dilution ratios and measured at 595 nm. The result was recorded in mg g^− 1^ FW.

#### Proline determination

The amount of proline was measured by homogenizing 0.2 g of leaf fresh weight in 2 mL of 3% aqueous sulfosalicylic acid and then centrifuged at 10000 rpm for 30 min. The supernatant was removed, and the pellet was washed with 3% aqueous sulfosalicylic acid twice. The supernatant was pooled, and the amount of proline was evaluated using ninhydrin reagent and toluene extraction [73]. The protocol for each determination was calibrated with the standard curve of proline solution within the detection range of the method (0-39 μg mL^− 1^).

#### Analysis of antioxidant enzyme activities

One gram of the leaf samples was weighted and quickly homogenized in 5 mL of 50 mM K–phosphate buffer (pH 7.0) and brought to 5 mM Na–ascorbate and 0.2 mM EDTA by the addition of concentrated stocks. The homogenate samples were centrifuged at 10000 rpm for 15 min at 4 °C. Then, the supernatants were used for enzyme assays carried out at 4 °C. The activity of SOD, POD, and CAT was measured, as previously established by Li et al. [74]. Fresh leaf samples (0.5 g FW) were chosen from 2-week-old treated explants, harvested, and ground in liquid nitrogen and extracted with the following method: 100 mM potassium phosphate buffer (pH 7.8) including 0.1 mM EDTA, 1% (w/v) PVP and 0.1% (v/v) Triton × 100. The extracted solution was centrifuged at 10,000 rpm for 15 min at 4 °C. The supernatant was collected and used to measure the activity of the enzymes. The GPX activity was assayed by monitoring the increase in absorbance at 470 nm (ε = 26.6 mM ^−1^cm ^-1^) during the polymerization of guaiacol. One unit of activity was defined as the amount of enzyme producing 1 μmol of tetraguaiacol per min at 25 °C.

### Statistical analysis

The experiment was conducted as a factorial experiment based on a completely randomized design with three replications and five explants in each plot. Data were statistically analyzed by MSTAT-C software and the means were compared using the LSD method at the level of 5 % error probability.

## Data Availability

The datasets used and/or analysed during the current study available from the corresponding author on reasonable request.

## Abbreviations

SOD: Superoxide dismutase
POD: Peroxidase
CAT: Catalase
Chl: Chlorophyll
ChlT: Total chlorophyll
CARs: Carotenoids
MDA: Malondialdehyde
*Fv*: Variable fluorescence
*Fm*: Maximal fluorescence from dark-adapted leaf
*Fv*/*Fm*: Maximum PSII
ROS: Reactive oxygen species
SiO_2_-NPs: Silicon dioxide nanoparticles
H_2_O_2_: Hydrogen peroxide
MSI: Membrane stability index
PEG: Polyethylene glycol
RWC: Relative water content
EC: Electrical conductivity
